# Effects of 2,2′-Azobis(2-methylpropionamidine) Dihydrochloride Stress on the Gel Properties of Duck Myofibrillar Protein Isolate

**DOI:** 10.3390/molecules28186721

**Published:** 2023-09-20

**Authors:** Xueshen Zhu, Jin Zhang, Xinyu Zhang, Qun Dai, Qingquan Fu

**Affiliations:** 1 Key Laboratory of Biological Functional Molecules of Jiangsu Province, College of Life Science and Chemistry, Jiangsu Second Normal University, Nanjing 211200, China; zj_zstu0918@126.com (J.Z.); zhangxinyu010616@163.com (X.Z.); njdaiqun@163.com (Q.D.); 2 School of Food Science, Nanjing Xiaozhuang University, Nanjing 211171, China

**Keywords:** AAPH, protein oxidation, myofibrillar protein, gel properties

## Abstract

The aim of this study was to investigate the biochemical properties and gel-forming capacity of duck myofibrillar proteins under the effects of 2,2′-azobis(2-methylpropionamidine) dihydrochloride (AAPH)-mediated oxidation. Duck myofibrillar proteins were extracted and treated with different concentrations of AAPH solutions (0, 1, 3, 5, 10 mmol/L) and then analysed for carbonyl content, dynamic rheology, protein profiles and gel-forming properties (colour, water holding capacity, gel strength and microstructure). The results showed that with increasing AAPH concentration, the carbonyl content of the proteins exhibited an increasing trend (*p* < 0.05); SDS-PAGE pattern changes indicated that moderate oxidation (3 mmol/L AAPH) induced myosin aggregation via covalent bonds including disulfide, enhanced protein–protein interactions, and thus affected the gel strength of the DMPs’ heat-induced gels. However, high oxidation (5 and 10 mmol/L AAPH) led to the partial degradation of the myosin heavy chain (MHC) isoforms, as evidenced by lower storage modulus and irregular microstructures, which significantly reduced gelation ability. These results suggest that the internal relationship between alkylperoxyl radical-induced oxidation should be taken into account in the processing of duck meat, as mild protein oxidation is conducive to improving gel quality.

## 1. Introduction

For a long time, the oxidation of lipids and the role of micro-organisms have been considered important factors in the deterioration of meat product quality. In recent years, the influence of protein oxidation on the quality of meat products has been gradually recognized [1,2]. Muscle protein could improve the tissue structure of meat products, in particular its gelling properties, thus playing an important role in the quality and sensory performance of meat products [3]. However, the substance of myofibrillar proteins is very sensitive to reactive oxygen free radicals. The oxidation of meat is inevitable during processing and storage. In addition, oxidative damage can alter the physical, chemical and functional properties of proteins, thus reducing the sensory and nutritional quality of the products [4]. Under the action of multi-layer metal ions, the oxidation of myofibrillar proteins can lead to the formation of protein carbonyls, which greatly reduces its gel-forming properties [5]. At the same time, Liu et al. found that mild oxidation can promote the cross-linking of disulfide bonds between protein molecules, thus making the protein network structure more stable [6]. Our previous papers mainly investigated the effects of oxidation modification on the gel-forming capacities of duck myofibrillar proteins (DMPs). We revealed that excessive oxidation led to the explicit cross-linking of DMPs, which negatively affected the gel-forming capacities of DMPs by hydroxy radicals and malondialdehyde. However, the moderate oxidation of malondialdehyde was beneficial for improving its gel-forming properties [7,8]. 2,2′-azobis(2-methylpropionamidine) dihydrochloride (AAPH) generates alkylperoxyl radicals upon degradation, which preferentially oxidize their main targets, tryptophan, cysteine, methionine, tyrosine and, to a lesser extent, histidine residues, respectively, thereby causing protein oxidation [9,10]. In general, the actual meat product system is composed of protein and lipids. When lipids are rapidly oxidized, proteins are subsequently susceptible to oxidation as promoted by lipid-derived radicals and hydroperoxides [11]. However, to our knowledge, discussion in the literature concerning the oxidation effect of alkylperoxyl radicals on the gel-forming capacity of duck myofibrillar proteins has been limited. Some recent studies have shown the effect of alkylperoxyl radicals on the oxidation of porcine myofibrillar protein [12] and casein [13]. The partial main chain breakage of proteins was also found. In this study, AAPH was used as a typical free radical intermediate for the oxidation of lipids [14]. Its effects on the different degrees of oxidation status (carbonyl, cross-linking) and the properties of the DMPs’ heat-induced gels, including their rheological characteristics and textures, were investigated, and their internal correlation was also discussed. The aim was to provide a theoretical basis to lay a foundation for the further control of the oxidation levels of duck meat and the rational use of oxidants.

## 2. Results and Discussion

### 2.1. Total Carbonyl Content

**Figure 1:**
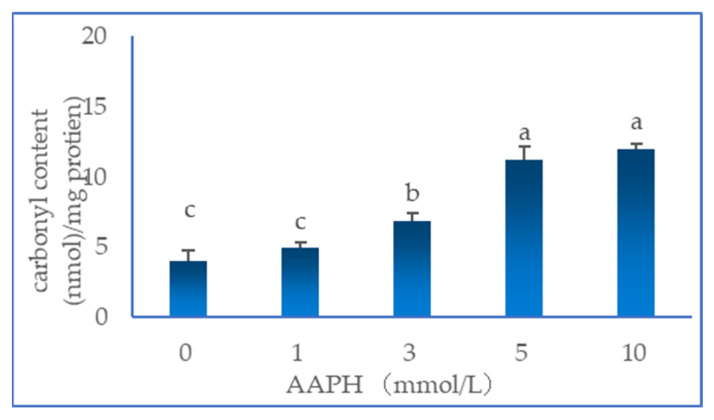
Effect of AAPH-mediated oxidation (0, 1, 3, 5 and 10 mmol/L) on carbonyl content of duck myofibrillar proteins. Means without a common superscript (abc) differ; *p* < 0.05.

As shown in Figure 1, with increasing AAPH concentration, the protein carbonyl content tended to increase gradually, reaching a maximum of 12.0 nmol/mg protein at 10 mmol/L AAPH. In general, the most reactive radicals tend to be the least selective. Thus, all side chain sites are oxidised to a greater or lesser extent by hydroxyl radicals [10]. A recent study has also reported that AAPH thermolysis induces casein crosslinking via the formation of Schiff bases between carbonyl groups (a known oxidation product of N-terminal amino groups and lysine residues) [13]. The direct oxidation of glutamyl or prolyl side chains by peroxyl radicals could also break peptide bonds, subsequently leading to peptide cleavage as a result of the α-amidation pathway or the formation of 2-pyrrolidone [15]. The cleavage of backbones is considered a major mechanism of protein carbonylation [16]. Therefore, oxidative damage to proteins can be reflected by carbonyl derivatives [17,18].

### 2.2. SDS-PAGE Pattern

**Figure 2:**
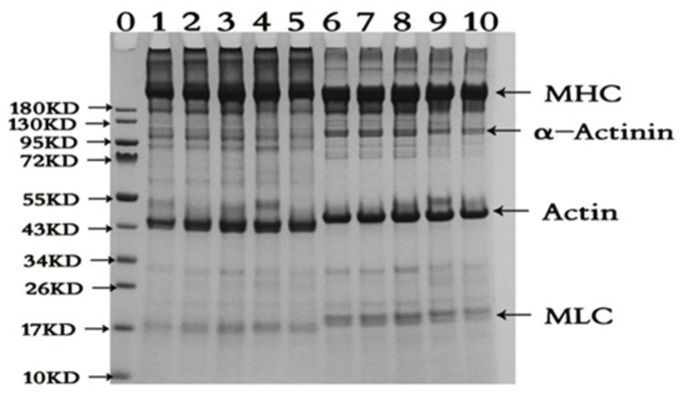
Results of gel electrophoresis (SDS-PAGE) of duck myofibrillar proteins at different concentrations of AAPH (0, 1, 3, 5 and 10 mmol/L). Note: 0 is marker; 1–5 are without DTT, and AAPH concentrations are 0, 1, 3, 5 and 10 mmol/L; 6–10 are with DTT and AAPH concentrations are 0, 1, 3, 5 and 10 mmol/L. MHC: myosin heavy chain, actin: actin, MLC: myosin light chain.

Oxidation cross-links proteins and simultaneously produces a variety of polymers, and the cross-linking is mostly associated with the myosin [19]. As shown in Figure 2, the concentration of AAPH has a certain effect on the structural composition of the proteins. When the AAPH concentration is 0, 1 and 3 mmol/L, it can promote the cross-linking of proteins and generate protein polymers. When the AAPH concentration is 5–10 mmol/L, it can partially degrade protein components. In the case of non-reducing conditions (-DTT), because there are a large number of high molecular weight myosin cross-links in the DMPs, the proteins are clustered in large quantities in an upper layer of stacking gel; it can be speculated that the proteins are attacked by alkylperoxyl radicals, causing the sulfhydryl group to form a disulfide bond and other covalent bonds, leading to the cross-linking of the proteins, thereby increasing its molecular weight and aggregation. Furthermore, the band intensity of the myosin heavy chain (MHC) cross-links first increased and then decreased as the AAPH concentration increased, reaching a maximum at 3 mmol/L AAPH concentration, and then decreased as the AAPH concentration increased. It is notable that myofibrillar proteins have a large number of sulfhydryl groups, among which myosin alone contains 42 sulfhydryl groups [20], which are easily attacked by reactive oxygen species to form disulphide bonds, leading to changes in protein structure and further affecting protein function. This stage is a reversible oxidation reaction, whereas irreversible oxidation reactions can also produce sulfinic acid and sulfonic acid [21]. Under the reducing condition (+DDT), the high molecular weight protein almost completely disappeared in an upper layer of stacking gel, and the intensity of the MHC and actin increased, indicating that disulfide bonds play an important role in protein cross-linking, and MHC and actin proteins are the main proteins that undergo cross-linking and aggregation during oxidation [22]. Around 17 kD, the samples without DTT have clear bands of myosin light chain 3 and fuzzy bands of myosin light chain 2, whereas in the reduced condition, the intensity of the myosin light chain 3 band of the samples decreases and myosin light chain 2 could be clearly seen. At the same time, a higher concentration of AAPH could induce the aggregation of myosin and the denaturation of myosin, troponin and tropomyosin, and lead to the partial degradation of the proteins [23]. In particular, both the actin and myosin light chains interacted with other peptides. This resulted in an increase in molecular weight, as indicated by the migration site. As reported, this means that the alkylperoxyl radicals would induce the formation of secondary free radicals which self-react to produce inter- and intra-molecular covalent bonds. The formation of ditryptophan bonds has also been implicated in the oxidative cross-linking of proteins. It is known that carbonyl groups undergo secondary reactions with basic residues (N-terminal amino groups) to generate Schiff bases, thereby inducing protein cross-linking [24].

### 2.3. Dynamic Rheological Properties

**Figure 3:**
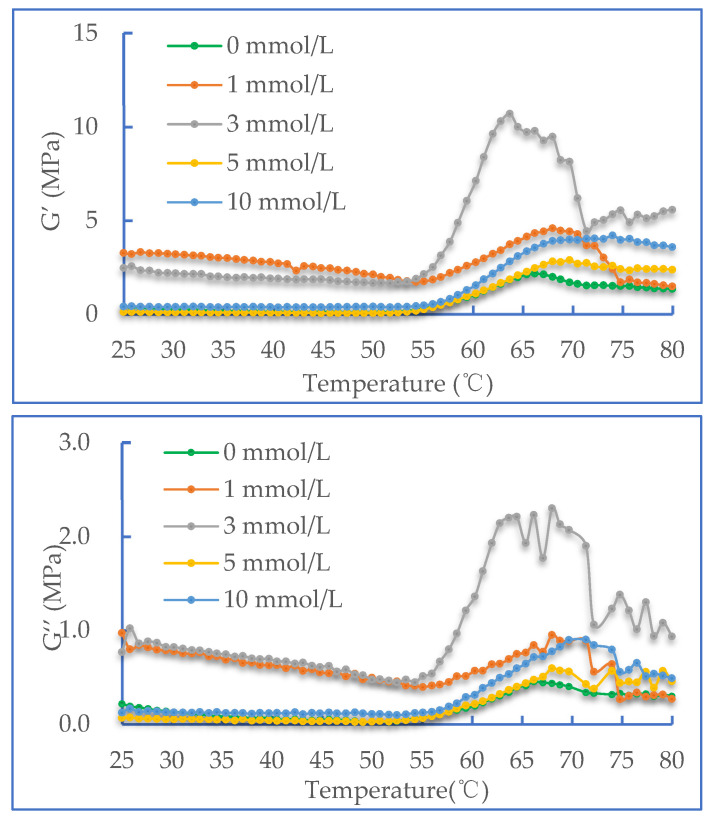
Change in the storage modulus (G’) of duck myofibrillar proteins at different concentrations of AAPH (0, 1, 3, 5 and 10 mmol/L).

From Figure 3, it can be seen that the change in the rheological properties of DMP oxidation is also related to the concentration of AAPH. At the end of the heating phase, the storage modulus of the protein increases with the increasing AAPH concentration (0–3 mmol/L). Meanwhile, the trend of the loss modulus is similar to that of the storage modulus. At the same time, at the heating stage, the value of the storage modulus is always higher than that of the loss modulus at the same temperature. The research of Feng and Xiong et al. [25] showed that at a high temperature of 65 °C, the denaturation of myosin causes the expansion of the structure, and the activated groups are also exposed. This change promotes the cross-linking of the proteins, which improves the viscoelasticity of the colloid and increases the storage modulus (G’). It was noteworthy that according to the analysis of SDS-PAGE in Section 2.2, after treatment with a higher concentration of AAPH, the MHC was degraded to a certain extent [26]. Among the proteins, the change range of protein G’, 3 mmol/L of AAPH oxidation treatment, was steeper and higher than those of the other concentrations of AAPH oxidation treatment. Simultaneously, the decrease in myosin thermal stability was caused by the partial degradation of the MHC, which was in turn caused by the high concentration oxidation. This led to the denaturation of myosin at the later stage of heating, which remarkably reduced the G’ of the system. Apparently, moderate oxidation is conducive to improving the strength of the protein gel to some extent. A significant amount of protein aggregation is caused by protein oxidation. Protein aggregation or a change in protein structure will have a major effect on its functional properties, and the most obvious is the change in the gel strength. It is noteworthy that for a sample to which 3 mmoL of AAPH was added, the gelation peak shifted forward to 63 ℃ compared to other groups. We speculate that mild protein oxidation could help the heat inducing self-aggregation of the tail region of myosin molecule, resulting in an impact on the start of the gelation process [27,28].

### 2.4. Gel Strength

**Figure 4:**
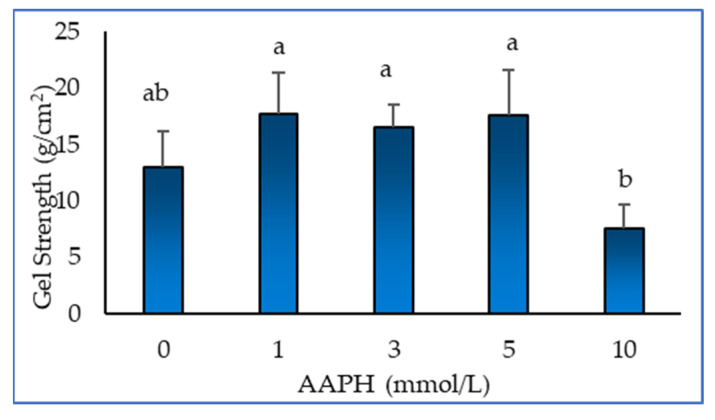
Effect of AAPH-mediated oxidation (0, 1, 3, 5 and 10 mmol/L) on gel strength of duck myofibrillar proteins. Means without a common superscript (ab) differ; *p* < 0.05.

Figure 4 shows the effect of oxidation on the gel strength of myofibrillar protein gel. It can be seen from the figure that the strength of the myofibrillar protein gel first subtly increases and then decreases. This shows that protein oxidation can improve the gel strength of myofibrillar protein to a certain extent. In general, the higher the gel strength, the denser, stronger and more stable the structure of the gel. Xiong [29] has shown that myofibrillar proteins can easily form a protein gel under thermal action after low concentration oxidation treatment, and the strength of the gel is increased due to the interaction between the proteins. Utrera et al. [30] showed that protein carbonylation caused by oxidation also changes the gel properties of the protein. According to the results of SDS-PAGE, high concentration oxidation treatment will reduce the polymerisation ability and brittleness of the protein gel.

### 2.5. Whiteness and Water Holding Capacity of Gel

**Figure 5:**
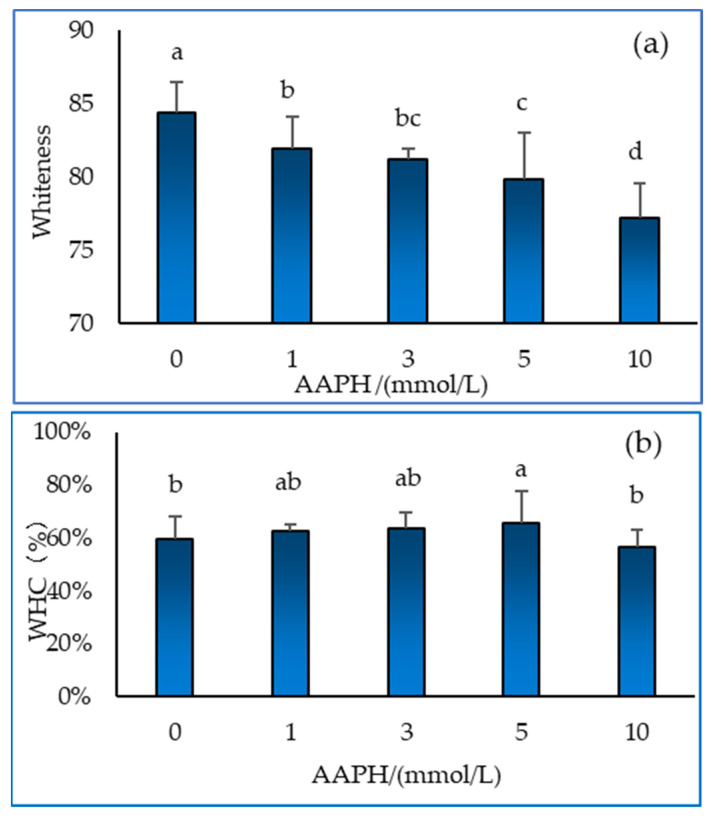
Effect of AAPH-mediated oxidation (0, 1, 3, 5 and 10 mmol/L) on gel whiteness (**a**) and water holding capacity (**b**) of duck myofibrillar protein gels. Means without a common superscript (abcd) differ; *p* < 0.05.

Protein denaturation affects the whiteness of the gel [31]. As shown in Figure 5a, the whiteness of the gel decreased significantly with increasing AAPH concentrations (*p* < 0.05). However, the whiteness tends to decrease when AAPH is added, which is not at all desired when processing. The water holding capacity (WHC) of the gel can reflect the roughness of its internal structure, which means that the gel could hold more water or prevent water seepage under different physical and chemical conditions. As can be seen from Figure 5b, as the AAPH concentration increases, the water holding capacity of the protein gel tends to first increase and then decrease. When the AAPH concentration is 4 mmol/L, the maximum water holding capacity is reached. When the AAPH concentration is 10 mmol/L, the water holding capacity of the gel decreases, which may be due to the degradation of protein components under high concentration oxidation, making it more difficult for gel to form. Lund et al. [32] have indicated the consequences of protein oxidation in muscle food have often been associated with changes in solubility and protein functionality, such as gelation and emulsifying properties, or WHC.

### 2.6. Water Status in Gel

**Figure 6:**
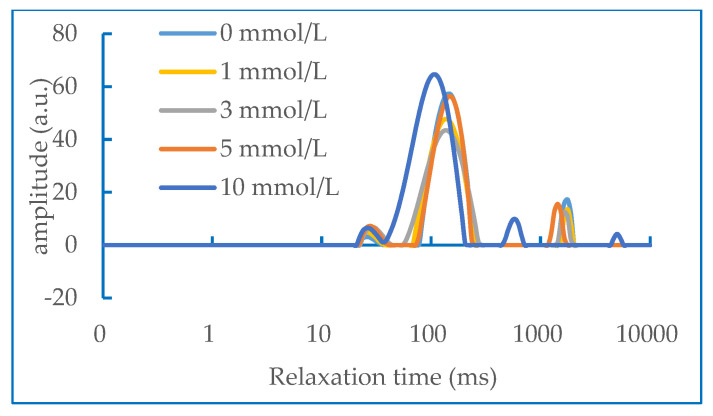
NMR characterisation of duck myofibrillar protein gels at different concentrations of AAPH (0, 1, 3, 5 and 10 mmol/L).

It can be seen from Figure 6 that the concentration of AAPH has an effect on the NMR relaxation time of the DMPs’ heat-induced gel. Han Minyi et al. [33] previously reported that there were generally four types of peaks in the NMR relaxation curve of myofibrillar protein gel after fitting, which corresponded to the states of four kinds of water, including bound water (T_2b_), moderately immobilized water (T_21_), immobilized water (T_22_), and free water (T_23_). The second peak and the third peak were collectively classified as water. In our study, it can be seen from the figure that the T_2_ relaxation intensity has a peak of moderately immobilized water between 10–95 Ms (T_21_), a peak of immobilized water between 100–1000 Ms (T_22_) and a peak of free water after 1000 Ms (T_23_). The peak with the largest area in the figure corresponds to immobilized water, indicating that myofibrillar proteins cross-link together when heated to form a network structure that binds a large amount of water molecules. When the concentration of AAPH is relatively low (0–3 mmol/L), the area of free water gradually decreases, whereas the result is the opposite when the concentration of AAPH is higher than 5 mmol/L. These results showed the same trend as the previous WHC results. Li Yin et al. combined the traditional drying method with the low field NMR technology and proved that the free water content is inversely proportional to the water holding capacity of the gel [34]. 

### 2.7. Gel Microstructure

**Figure 7:**
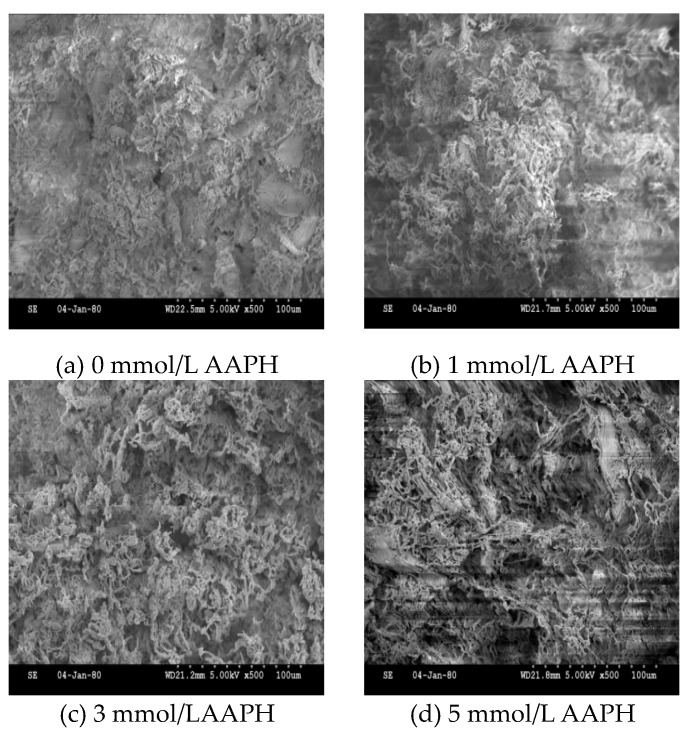

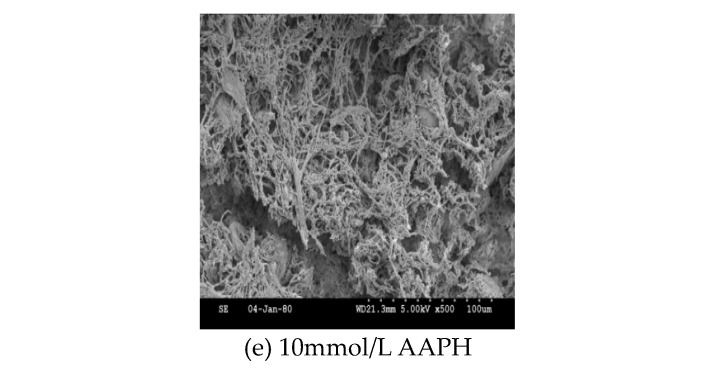
Gel microstructure analysis of duck myofibrillar proteins treated with AAPH concentrations of 0 mmol/L (**a**), 1 mmol/L (**b**), 3 mmol/L (**c**), 5 mmol/L (**d**), and 10 mmol/L (**e**).

The microstructure of the protein gel is an important parameter for studying its structure and properties. The SEM image of the protein gel treated with 4% glutaraldehyde is shown in Figure 7. The results show that the internal structure of the gel changed significantly after AAPH oxidised the protein. Proper oxidation can promote protein cross-linking, thus encouraging irregular aggregates to participate in the gel [35], improving the network structure. As shown in Figure 7, the structure of the DMPs’ gel deteriorates as the AAPH concentration increases and the degree of deterioration also increases. When the concentration of AAPH is 10 mmol/L, the micropores continue to expand and break, forming fibre fragments, and the gel structure begins to collapse as irregular cracks appear.

## 3. Materials and Methods

### 3.1. Sample Preparation and Reagents

Duck breasts, obtained from a local market in Lishui District, Nanjing, Jiangsu Province, were taken to the lab at 4 °C, then divided into 30 g per sealed bag and stored at −80 °C before use. The reagents, including 2,2-Azobis (2-methylpropionamidine) dihydrochloride, were obtained from Shanghai Macklin Biochemical Co., Ltd., Shanghai, China, and are analytically pure.

### 3.2. Extraction of Duck Myofibrillar Proteins (DMPs)

Duck myofibrillar proteins were extracted according to the process detailed by Zhu et al. [36] Briefly, the −80 °C frozen duck breasts were taken out and placed into centrifuge tubes, and 5 times the volume of extraction solution was added (100 mmol/L NaCl, 2 mmol/L MgCl_2_, 1 mmol/L EGTA, 10 mmol/L K_2_HPO_4_, pH 7.0) and then centrifuged at 4 °C, 4000× *g* for 10 min. The supernatant was then discarded. The above steps were repeated three times, and the solution was also filtered through two layers of sterilized gauze in the meantime. Subsequently, the precipitation was homogenised with 5 times the volume of the extract (12.5 mmol/L NaCl, 2.5 mmol/L MgCl_2_, 1. 25 mmol/L EGTA, 12.5 mmol/L K_2_HPO_4_, 1% Triton X-100, pH 7.0), then centrifuged at 4 °C 4000× *g* for 10 min to remove the membrane protein; finally, 4 times the volume of 0. 1 mol/L NaCl was added, then centrifuged at 4 °C, 4000× *g*, for 10 min. The final precipitate was collected to obtain duck myofibrillar proteins.

### 3.3. Oxidation of Duck Myofibrillar Proteins

The oxidation of duck myofibrillar proteins was carried out according to the procedures detailed by Zhou et al. [12] with slight modifications. Briefly, the protein content of the DMPs was firstly measured using the biuret method and then diluted to 30 mg/mL. The oxidation solution for the duck myofibrillar proteins was prepared according to Table 1, then incubated for 24 h at 4 °C and shaken while avoiding light. After oxidation, the DMPs were immediately centrifuged at 4 °C 8000× *g* for 10 min. The precipitate was washed twice with an appropriate volume of distilled water and centrifuged again under the same conditions. The supernatant was then discarded, and the precipitate was considered a sample of oxidised myofibrillar protein.

### 3.4. Carbonyl Content

The carbonyl content of the protein was determined according to the 2,4-dinitrophenylhydrazine colour development method used by Soglia et al. [37] with slight modifications. In brief, the carbonyl content is expressed in nmol/mg protein in terms of the molar extinction coefficient 22,000 L/(mol·cm), then calculated according to Equation (1):

(1) $$\mathrm{Carbonyl}\;\mathrm{content}\;\left(\mathrm{nmol}/\mathrm{mg}\;\mathrm{protein}\right)=\frac{\left[A_{370}-A_{370\left(\mathrm{blank}\right)}\right]\times 10^{6}}{22,000\times \left[A_{280}-\left(A_{370}-A_{370\left(\mathrm{blank}\right)}\right)\times 0.43\right]}$$

### 3.5. Sodium Dodecyl Sulfonate Polyacrylamide Gel Electrophoresis Analysis (SDS-PAGE)

SDS-PAGE was performed according to the method described by Jia et al. [38] with slight modifications. The concentration of the protein solution was adjusted to 2 mg/mL, and mixed with an equal amount of 2 × standard SDS sample loading buffer, with or without dithiothreitol (DTT), and heated for 3 min in a dry heater at 100 °C. SDS-PAGE electrophoresis ran at 220 V for 45 min using NuPAGE^TM^ 5–12% Bis-Tris gel (NuPAGE^TM^, Invitrogen, Carlsbad, CA, USA). The gel was then stained with Coomassie brilliant blue G-250 before conducting analyses.

### 3.6. Dynamic Rheological Test

The protein solution was measured using a rheometer (MCR-301, Anton Paar, Graz, Austria) in oscillatory mode as described by Zhuang et al. [39]. Brief parameters were set as follows: a 50 mm flat plate with a gap of 1 mm, a frequency of 0.1 Hz, a strain of 2%, a temperature rise from 25 °C to 85 °C at a rate of 2 °C / min, and a temperature fall rate of 5 °C / min. The storage modulus (G’) and loss modulus (G’’) were then recorded.

### 3.7. Gel Preparation

According to the method described by Xia et al. [40], the protein solution (40 mg/mL) was heated at 80 °C for 35 min to induce gelation, then cooled with ice water for 30 min, and stored 4 °C to equilibrate overnight.

### 3.8. Gel Strength Measurement

The strength of the gel was measured using the texture analyser (TAXT plus Plaser, Stable Micro Systems, Godalming, UK). The following parameters were used: a P/0.5r probe, a 0.5 mm/s rate, a 0.5 mm/s test rate, a 0.5 mm/s post-test rate, a 5 mm probe depth distance and a trigger force of 4 g, and repeat each treatment sample 3 times.

### 3.9. Gel Whiteness and Water Holding Capacity 

The whiteness of the gel was measured using Minolta CR-400 (illuminant D65) equipment (Minolta Camera, Osaka, Japan) calibrated with a standard white board. A C light source was used as the light source for the measurement, and the L*, a* and b* of the gel were recorded. The whiteness of the gel is calculated according to methods used by Salvador et al. [41] as shown in Equation (2):

(2) $$Whiteness=100-\sqrt{{\left(100-L^{*}\right)}^{2}+a^{*}{}^{2}+b^{*}{}^{2}}$$

The water holding capacity (WHC) of the gel was determined using centrifugation methods according to Zhu et al. [42]. 

### 3.10. Water Status in Gel 

The T_2_ relaxation time of the water in the samples was measured using an NMR analyser (MesoMR23-060H-1, Niumag Electric Co., Shanghai, China) according to methods described by Xia et al. [43]. Firstly, a standard oil sample was calibrated, then about 2 g of the sample was placed in a centrifuge tube inside the instrument, and then the CPMG sequence was selected according to the spin–spin relaxation time. The proton resonance frequency was set at 22.6 MHz and the measurement was performed at 32 °C. The following parameters were set: the number of repeated samples is 4, the waiting time is 2000 ms, the number of echoes is 9000.

### 3.11. Gel Microstructure

The gel samples were first cut into cubes (3 × 3 × 3 mm^3^), fixed with 4% glutaraldehyde, then observed using a Hitachi S-3000N scanning electron microscope (Tokyo, Japan) at an accelerating voltage of 20 kV.

### 3.12. Statistical Analysis

SPSS 19.0 (version 20, SPSS Inc., Chicago, IL, USA) was used for one-way ANOVA, and Duncan’s multiple comparison method was used for statistical analysis (*p* < 0.05 indicates significant difference).

## 4. Conclusions

As the concentration of AAPH increases, the carbonyl group content of the duck myofibrillar proteins increases. At relatively low concentrations of AAPH (1–3 mmol/L), the protein is attacked by peroxy radicals, which cross-link the protein, increase its molecular weight and form aggregates. Under the action of high concentrations of AAPH (5–10 mmol/L), protein components are partially degraded. In particular, at 10 mmol/L AAPH, the gel structure begins to collapse and large irregular cracks appear, making gel formation more difficult. It can be seen that the alkylperoxyl radical system has an obvious effect on the gel properties of duck myofibrils. It is noteworthy that a mild concentration of AAPH (3 mmol/L) can effectively improve the texture properties and water retention capacity of the gel.

## Figures and Tables

**Table 1:** Preparation of oxidation solution for myofibrillar proteins.

AAPH Concentraton (mmol/L)	0	1	3	5	10
DMPs solution (mL)	6				
AAPH stock solution (mL)	0	0.2	0.6	1	2
20 mmol/L, pH 6.5 PBS (mL)	4	3.8	3.4	3	2

Note: AAPH stock solution concentration is 50 mmol/L.

## Data Availability

Not applicable.

## Sample Availability

Samples of the compounds 2,2′-azobis(2-methylpropionamidine) dihydrochloride are available from the authors.
